# Gabor filter-based statistical features for ADHD detection

**DOI:** 10.3389/fnhum.2024.1369862

**Published:** 2024-04-10

**Authors:** E. Sathiya, T. D. Rao, T. Sunil Kumar

**Affiliations:** ^1^Division of Mathematics, Vellore Institute of Technology, Chennai, India; ^2^Department of Electrical Engineering, Mathematics and Science, University of Gävle, Gavle, Sweden

**Keywords:** attention deficit/hyperactivity disorder, Gabor filter, EEG classification, ADHD, morphological

## Abstract

Attention deficit/hyperactivity disorder (ADHD) is a neuropsychological disorder that occurs in children and is characterized by inattention, impulsivity, and hyperactivity. Early and accurate diagnosis of ADHD is very important for effective intervention. The aim of this study is to develop a computer-aided approach to detecting ADHD using electroencephalogram (EEG) signals. Specifically, we explore a Gabor filter-based statistical features approach for the classification of EEG signals into ADHD and healthy control (HC). The EEG signal is processed by a bank of Gabor filters to obtain narrow-band signals. Subsequently, a set of statistical features is extracted. The computed features are then subjected to feature selection. Finally, the obtained feature vector is given to a classifier to detect ADHD and HC. Our approach achieves the highest classification accuracy of 96.4% on a publicly available dataset. Furthermore, our approach demonstrates better classification accuracy than the existing methods.

## 1 Introduction

On a global scale, it is estimated that approximately 5% of children are affected by attention deficit hyperactivity disorder (ADHD) (Song et al., 2021), one of the most common heterogenous disorders affecting children, characterized by inattention, impulsiveness, and hyperactivity. Children with ADHD have an adverse impact behavioral patterns, particularly in education and interpersonal growth, it may even extend into adulthood (Altınkaynak et al., 2020). According to Xu et al. (2018) and TaghiBeyglou et al. (2022), individuals with ADHD spanning from childhood to adulthood often experience challenges in psychosocial and neuropsychological functioning. Untreated ADHD leads to worse social and professional functioning, a larger chance of comorbid, and a higher risk of serious depressive and anxiety disorders (American Psychiatric Association, 2013). Therefore, early detection and timely therapeutic intervention are of essential importance in preventing the severity of ADHD in children.

Traditionally, the diagnostic assessment of ADHD in children is conducted by psychiatrists through interviews with parents and/or the child. Manual diagnosis can be subjective, and this evaluation process is often time-consuming, demands a high level of medical expertise, and can be prone to error in certain cases (Khare and Acharya, 2023). In recent years, quantitative techniques such as brain signaling examinations have been conducted to establish a diagnosis.

Researchers have been utilizing many neuroimaging techniques to diagnose ADHD, some of them are magnetoencephalography (MEG) (Hamedi et al., 2022), magnetic resonance imaging (MRI) (Zhou et al., 2021), and electroencephalogram (EEG) (Allahverdy et al., 2016). However, some of these approaches, such as MEG, are radioactive, bulky, and costly (Khare and Acharya, 2023). On the other hand, EEG signals are portable and cost-effective solutions for ADHD detection (Maniruzzaman et al., 2023), and they have also been used in various applications (Kumar et al., 2015; Khare and Bajaj, 2020).

Over the last decade, researchers have extracted various linear, non-linear, and morphological features from time (Yang et al., 2016; Khaleghi et al., 2020; Maniruzzaman et al., 2023), frequency (Mueller et al., 2010; Kaur et al., 2019; Khaleghi et al., 2020), and time-frequency (Öztoprak et al., 2017; Altınkaynak et al., 2020; Joy et al., 2022) domain-based methodologies. Altınkaynak et al. (2020) utilized event-related potentials (ERPs), while participants engaged in an auditory oddball task, which resulted in longer P300 latency for ADHD patients and smaller P300 amplitude for healthy control (HC). Maniruzzaman et al. (2023) performed a channel selection method and extracted various times, morphological, and non-linear features for the classification of ADHD and HC. The approach in Khaleghi et al. (2020) extracted various morphological, non-linear, time, frequency, and time–frequency-based features; among these non-linear features (Petrosian and Katz fractal dimensions, Lyapunov exponent, approximate entropy, and Lempel-Ziv complexity) extracted from EEG, provides a good quantitative tool in the detection of ADHD. Similarly, Kaur et al. (2019) extracted time-domain features, namely morphological, complexity features (power of scale-freeness and graph index complexity), and frequency-domain features such as Katz and Higuchi algorithm for diagnosis. Chow et al. (2019) developed an approach based on Hjorth mobility (M), and the results indicated that M values in the control group were significantly higher than the ADHD individuals. In the frequency domain, the power of different EEG frequency bands was used to diagnose ADHD (Altınkaynak et al., 2020). It indicates increased theta power and a higher theta/beta ratio in ADHD patients compared to HC, but the use of non-linear features outperformed frequency band features (González, 2022). Altınkaynak et al. (2020) investigated the entropy of the discrete wavelet transform (DWT) of auditory evoked potentials for the classification between ADHD and HC, and it exhibited significantly different values in both groups. Similarly, Castro-Ospina et al. (2012) investigated the occurrence of low-frequency bands computed through wavelets and empirical mode decomposition (EMD) to find the differences in the patterns of ERP waves between ADHD patients and control subjects. Tor et al. (2021) computed autoregressive modeling coefficients and relative wavelet energy from EMD and DWT for the detection of ADHD.

Since the EEG signal is characterized by non-stationary behavior and a diverse range of time-frequency components, using Gabor filters can be an advantage for discovering the signal's descriptive features. In recent years, researchers have prominently used Gabor filters in image processing (Hu et al., 2020), and computer vision-based applications (Oppong et al., 2022). In addition, Gabor filter-based features have been found to be effective in signal classification tasks (Kumar et al., 2015) and even integrated into deep learning models (Barshooi and Amirkhani, 2022; Hammouche et al., 2022; Khalifa et al., 2022; Oppong et al., 2022). Despite these advantages, the potential of the Gabor filter has not been explored for ADHD detection. Therefore, in this paper, we explore the bank of Gabor filters for ADHD detection.

To the best of our knowledge, there has been no prior investigation that evaluated Gabor filter-based features for the classification of EEG signals into ADHD and HC.

The following contributions made by our study are:

1. Explored Gabor-based statistical features for the classification of EEG signals into ADHD and HC.
2. Our approach has been validated using 10-fold cross-validation and an 80:20 train-test split.
3. Our approach has outperformed the existing approaches in the detection of ADHD.

This paper is organized as follows: Section II explains the experimental procedure for ADHD detection. In Section III, Results and Discussion is presented, and finally, the conclusions are provided in Section IV.

## 2 Methodology

**Figure 1:**
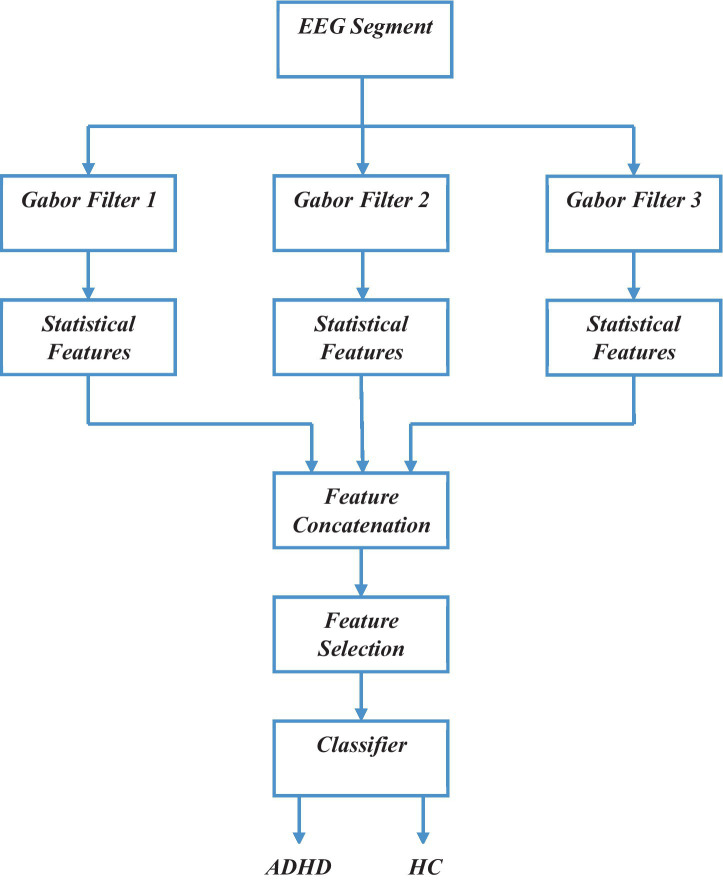
Block diagram of proposed Gabor filter-based ADHD detection approach.

In this section, Figure 1 represents the flowchart of the proposed approach. First, the EEG signals are processed using the bank of Gabor filters. This process converts a non-stationary EEG signal to a narrow-band signal. From each of those narrow-band signals, we extract a set of statistical features. Finally, the features extracted are concatenated and fed to classifiers to classify EEG signals into ADHD or HC. The description of each step is given below.

### 2.1 Gabor filters

The Gabor filter acts as a bandpass filter and provides good time-frequency localization (Gabor, 1946). Furthermore, using a bank of Gabor filters for the decomposition of signal makes it easier to extract discriminating information from a particular frequency range. The mathematical representation of the Gabor filter is as follows:


(1) $$g\left(t\right)=\frac{1}{\sqrt{2\pi \sigma^{2}}}\exp\left(-\frac{t^{2}}{2\sigma^{2}}+j2\pi f_{c}t\right)$$

where $f_{c}$ - central frequency,

*σ* – Standard deviation of the Gaussian function.

The response $z\left(t\right)$ is computed through the convolution of the input signal $y\left(t\right)$ with the Gabor function $g\left(t\right)$ as described by Equation (1). Finally, the magnitude of the Gabor response will be determined for feature extraction.

### 2.2 Statistical features

The feature extraction process is crucial in the classification process, as the choice of features significantly impacts the performance of the classification. Local binary pattern (LBP)-based histogram features are commonly used to extract features from the responses of Gabor filters (Kumar et al., 2015; Samiee et al., 2017; Sunil Kumar and Kanhangad, 2017; Kumar and Kanhangad, 2018). The length of the histogram (feature length) is 256. As we are conducting multichannel EEG signals (19 channels), extracting the traditional features will lead to a high-dimensional feature vector. Therefore, we have extracted four statistical features from each of the Gabor filter responses. In our study, statistical features such as entropy, standard deviation, skewness, and kurtosis were extracted from the magnitude of the response $z\left(t\right)$ . Mathematical equations for the aforementioned features can be found in Sunil Kumar and Kanhangad (2017).

### 2.3 Feature concatenation and classification

In this process, the feature vector is constructed through the concatenation of statistical features extracted from $z\left(t\right)$ across all the channels. To classify the EEG segment into ADHD and HC, the feature vector is fed into classifiers. In our approach, we have used two classifiers; namely support vector machine (SVM) (Maniruzzaman et al., 2023) and k-nearest neighbors (k-NN) (Altınkaynak et al., 2020).

### 2.4 Feature selection

Feature selection (FS) is important for improving the performance of predictive models by eliminating redundant elements in a dataset, thereby maintaining only the most important features. In our study, we explored the *t*-test (Maniruzzaman et al., 2023) and the chi-square test (Rangarajan and Mahanand, 2014) to decrease the length of the feature vector and improve the accuracy (Acc) of classification.

The algorithm of our proposed approach is given below.

#### 2.4.1 Algorithm of our proposed approach

**Step 1:** process the multichannel EEG segment with a bank of Gabor filters.

**Step 2:** compute statistical features from each of the Gabor responses.

**Step 3:** concatenate the features corresponding to each individual channel to get the final feature vector. Apply the FS technique to reduce the length of the feature vector.

**Step 4:** train the classification models, such as k-NN and SVM, and evaluate their performance.

## 3 Experimental results

This section presents a comprehensive description of the dataset, followed by Results and Discussion.

### 3.1 Dataset

In our study, a publicly available dataset (Nasrabadi et al., 2020) has been utilized for detecting ADHD from EEG signals. The EEG signals acquired in this dataset include 61 children diagnosed with ADHD and 60 HC, and all the participants were within the age range of 7 to 12 years. The diagnostic criteria for the ADHD group with confirmation, are based on psychiatric evaluation in accordance with DSM-IV guidelines (American Psychiatric Association, 2013). Notably, the ADHD children had received Ritalin treatment for up to 6 months. The control group was free of psychiatric disorders, epilepsy, and high-risk behaviors. EEG recordings were acquired according to the 10-20 standard, utilizing 19 channels, and a sampling frequency of 128 Hz. In our study, the EEG signal is divided into segments with a duration of 30 and 60 s. For further information about the dataset, refer Nasrabadi et al. (2020).

### 3.2 Results

To validate the performance of our approach, the following metrics are used namely, Acc, specificity (Sp), and sensitivity (Sn) in which Sp denotes the capacity to correctly categorize normal data, while Sn signifies the ability to identify ADHD-related events, whereas Acc is defined as the ratio of correctly classified segments to the total number of segments in the test set.

**Figure 2:**
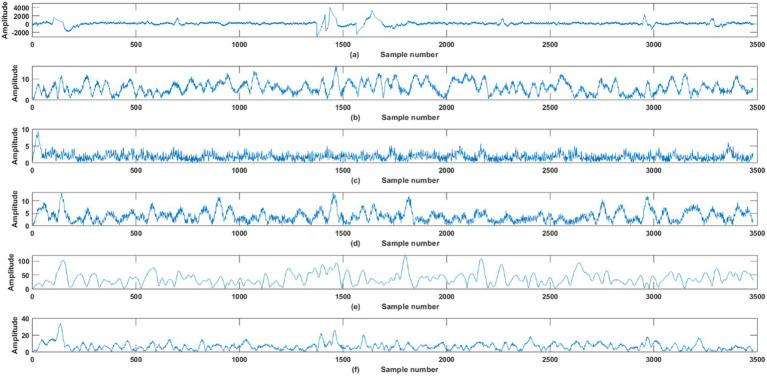
Plot of **(A)** Raw EEG signal. **(B–F)** Magnitude response of each of the Gabor filters in the filter bank.

In order to study the influence of the number of Gabor filters, we have performed our experiments by selecting the number of Gabor filters as 3, 4, and 5. The central frequency ( $f_{c}$ ) and standard deviation ( σ ) of the individual filters are selected to cover the range of frequencies extending from 1 to 64 Hz (half of the sampling frequency). These parameters are shown in Table 1. Figure 2 shows an exemplary plot of the raw EEG signals and magnitude response of the Bank of Gabor filters with five filters (the parameters involved in each of these Gabor filters is shown in Figure 2).

**Table 1:** Gabor filter parameters used in our approach.

No. of Gabor filters	Central frequency	Standard deviation
3	12.8, 25.6, 38.4	10, 10, 5
4	12.8, 25.6, 38.4, 51.2	10, 10, 10, 10
5	10.24, 20.48, 30.72, 40.96, 51.2	12, 12, 12, 12, 10

To show the effectiveness of our approach, we have performed two sets of experiments: in the first set, we conducted 10-fold cross-validation, and in the second set, we divided the entire dataset into 80:20 train-test data.

The performance metrics obtained are shown in Tables 2–7. More specifically, Tables 2–4 show the results obtained when an EEG segment of 30 s is used, while Tables 5–7 show when an EEG segment of 60 s is used for classification purposes.

**Table 2:** Performance metrics obtained with an EEG segment of 30 s and three Gabor filters.

Validation	Classifier	FS	Acc (%)	Sn (%)	Sp (%)	No. of features
10-fold	SVM	Without FS	86.1	83.26	88.25	228
		Chi-square	85.9	72.12	89.52	120
		*t*-test	84.1	79.18	87.93	80
	k-NN	Without FS	89.5	84.89	92.69	228
		Chi-square	91.6	88.57	93.96	120
		*t*-test	88	83.67	91.42	80
80:20	SVM	Without FS	77.7	80.61	90.47	228
		Chi-square	89.3	79.59	89.68	140
		*t*-test	81.2	73.46	86.50	83
	k-NN	Without FS	88.4	82.65	92.06	228
		Chi-square	92.9	86.73	93.25	140
		t-test	90.2	70.08	91.26	83

**Table 3:** Performance metrics obtained with an EEG segment of 30 s and four Gabor filters.

Validation	Classifier	FS	Acc (%)	Sn (%)	Sp (%)	No. of features
10-fold	SVM	Without FS	95.3	94.69	95.85	304
		Chi-square	93.7	92.24	94.90	85
		*t*-test	96.1	95.10	96.81	144
	k-NN	Without FS	93.4	91.02	95.22	304
		Chi-square	93.9	81.22	86.62	85
		*t*-test	95.5	94.28	96.49	144
80:20	SVM	Without FS	93.7	90.30	96.03	304
		Chi-square	95.5	91.83	94.44	85
		*t*-test	94.6	93.87	95.63	159
	k-NN	Without FS	93.7	89.29	94.04	304
		Chi-square	92.8	87.75	95.23	85
		t-test	**96.4**	**91.83**	**96.42**	**159**

The bolded values indicate the classifier models with the highest accuracy scores among the ones presented in each table.

**Table 4:** Performance metrics obtained with an EEG segment of 30 s and five Gabor filters.

Validation	Classifier	FS	Acc (%)	Sn (%)	Sp (%)	No. of features
10-fold	SVM	Without FS	95.2	93.46	96.49	380
		Chi-square	94.5	93.87	94.90	85
		*t*-test	95.5	93.46	97.13	148
	k-NN	Without FS	92.7	91.83	93.31	380
		Chi-square	93.4	89.79	96.17	85
		*t*-test	93.4	89.79	96.17	148
80:20	SVM	Without FS	94.6	88.77	95.23	380
		Chi-square	**96.4**	**89.79**	**92.85**	**85**
		*t*-test	88.4	75	73.71	79
	k-NN	Without FS	93.7	86.22	92.85	380
		Chi-square	91.9	89.79	94.44	85
		*t*-test	88.6	77.04	91.26	79

The bolded values indicate the classifier models with the highest accuracy scores among the ones presented in each table.

**Table 5:** Performance metrics obtained with an EEG segment of 60 s and three Gabor filters.

Validation	Classifier	FS	Acc (%)	Sn (%)	Sp (%)	No. of features
10-fold	SVM	Without FS	82.1	75	87.5	228
		Chi-square	81.7	76.04	85.95	45
		*t*-test	77.7	69.79	83.59	35
	k-NN	Without FS	76.8	68.75	82.81	228
		Chi-square	77.7	67.70	85.15	45
		*t*-test	86.2	79.16	91.04	35
80:20	SVM	Without FS	75	70.12	87.37	228
		Chi-square	75	67.53	87.37	45
		*t*-test	79.5	68.83	84.46	35
	k-NN	Without FS	86.4	59.74	81.55	228
		Chi-square	**90.9**	**67.53**	**86.04**	**45**
		*t*-test	88.6	67.53	91.26	35

The bolded values indicate the classifier models with the highest accuracy scores among the ones presented in each table.

**Table 6:** Performance metrics obtained with an EEG segment of 60 s and four Gabor filters.

Validation	Classifier	FS	Acc (%)	Sn (%)	Sp (%)	No. of features
10-fold	SVM	Without FS	85.3	80	89.23	304
		Chi-square	79.6	71.57	85.38	74
		*t*-test	86.2	81.05	90	100
	k-NN	Without FS	77.8	63.31	86.15	304
		Chi-square	80	71.57	86.15	74
		*t*-test	82.2	72.63	89.23	100
80:20	SVM	Without FS	84.4	76.31	84.61	304
		Chi-square	86.7	78.94	87.5	100
		*t*-test	88.9	80.26	87.5	76
	k-NN	Without FS	86.7	65.78	85.57	304
		Chi-square	75.6	77.63	87.5	100
		*t*-test	80	71.05	90.38	76

**Table 7:** Performance metrics obtained with an EEG segment of 60 s and five Gabor filters.

Validation	Classifier	FS	Acc (%)	Sn (%)	Sp (%)	No. of features
10-fold	SVM	Without FS	84.4	80	87.69	380
		Chi-square	80.4	73.73	84.61	60
		*t*-test	84	75.78	90	79
	k-NN	Without FS	78.7	67.36	86.92	380
		Chi-square	84	77.89	88.46	60
		*t*-test	85.8	76.84	92.30	79
80:20	SVM	Without FS	82.2	72.36	89.42	380
		Chi-square	86.7	67.73	85.57	50
		*t*-test	86.7	80.26	85.57	79
	k-NN	Without FS	77.8	59.21	86.53	380
		Chi-square	75.6	64.47	87.5	50
		*t*-test	86.7	67.10	82.69	79

Tables 2–4, it is evident that our approach performs better when the number of Gabor filters is set to 3 or 5, while the performance of the approach was comparatively inferior when the number of Gabor filters is set to 4 for 30-s duration. It can also be observed from our results that the classification improved when FS was included at the same time, and the number of features performed was reduced.

To understand the impact of segment length on the performance of the proposed approach, we performed the experiments by segmenting the EEG signals for 60 s. These results are shown in Tables 5–7. It can be observed from Tables 5–7 that the proposed approach achieved a maximum Acc of 90.9% when a 60-s segment is used. The performance is inferior when compared to the performance achieved with 30 s of EEG data.

### 3.3 Performance comparison

The performance comparison of our approach with existing approaches is shown in Table 8. Our approach has achieved better performance than the existing approaches in Chen et al. (2019), Altınkaynak et al. (2020), Ekhlasi et al. (2021), Kim et al. (2021), Parashar et al. (2021), Maniruzzaman et al. (2022), and Alim and Imtiaz (2023). The approaches in Chen et al. (2019), Altınkaynak et al. (2020), and Kim et al. (2021) have performed experiments on different datasets, while the approaches in Ekhlasi et al. (2021), Parashar et al. (2021), Maniruzzaman et al. (2022), and Alim and Imtiaz (2023) have performed experiments on the same dataset as ours. Chen et al. (2019) performed four distinct methods: relative spectral power, spectral power ratio, complexity analyses, and bicoherence for resting-state EEG feature extraction. The classifier constructed by selecting features from all four methods obtained an Acc of 85% on data acquired from 108 subjects. Kim et al. (2021) investigated the mismatch negativity (MMN) features, exploring both sensor-level attributes such as amplitude, latency, and source-level characteristics across various brain regions and achieved an Acc of 81%. It should be noted that authors have collected data from only 79 subjects. Altınkaynak et al. (2020) analyzed wavelet, non-linear (Higuchi algorithm), and morphological features (P300 latency and amplitude parameters) by using different classifiers and obtained the highest Acc of 91.3%. Parashar et al. (2021) used various combinations of channels from different brain regions (frontal, central, occipital, and parietal) that are directly fed to classifiers for classification purposes. When considering all channels of the right hemisphere, the authors reported an Acc of 84%. Alim and Imtiaz (2023) used EEG linear features from the four sub-bands and achieved an Acc of 94.2%. Ekhlasi et al. (2021) obtained the effective connectivity matrices (ECMs) of each individual by directed phase transfer entropy (dPTE) between each pair of electrodes, achieving an Acc of 89.7% with the selected features of the effective connectivity vector (ECV). Maniruzzaman et al. (2022) extracted morphological and time-domain features such as absolute amplitude, positive area, negative area, total area, peak-to-peak, mean, median, energy, power, standard deviation, skewness, kurtosis, coefficient of variation, H parameter activity, mobility, and complexity of EEG signals and obtained an Acc of 94.2%. However, our Gabor filter-based approach achieved the highest classification Acc of 96.4 %, outperforming the existing approaches. The superior performance of our approach is due to the efficacy of the Gabor filter in time-frequency domain localization (Gabor, 1946). Extracting features from the narrow-band signals (obtained after processing through the bank of Gabor filters) may lead to an effective time-frequency representation of EEG signals, which could be the possible reason for its superior performance. The key advantage of our approach is that it is simple yet effective for detecting ADHD. Whereas, the limitation of our approach is that the number of features increases as the number of Gabor filters increases.

**Table 8:** Performance comparison with existing approaches.

Author	Dataset	Feature extraction	Classifier	Acc
Chen et al. (2019)	108	Power spectral features are used with SVM for classification.	SVM	84.59%
Kim et al. (2021)	79	Mismatch negativity (MMN) features as biomarkers for classification.	–	81.0%
Altınkaynak et al. (2020)	46	Wavelet-based features	SVM, k-NN RF, AB, MLP, NB, LR	91.3%
Parashar et al. (2021)	120	Different combinations of the feature channels	AB, RF, SVM	84%
Alim and Imtiaz (2023)	120	PCA-based features to train a Gaussian SVM model.	SVM	94.2% (80:20)
Ekhlasi et al. (2021)	121	Directed Phase Transfer Entropy	ANN	89.7%
Maniruzzaman et al. (2022)	121	Morphological Time-domain	SVM, k-NN, MLP, LR	94.2%
**Present study**	121	Gabor filter-based features were employed as features with SVM and k-NN.	SVM, k-NN	**96.1% (80:20), 95.5% (10-fold).**

SVM: support vector machine; AB: Ada boost; RF: random forest; ANN: artificial neural network; k-NN: k-nearest neighbor; MLP: multilayer perceptron; LR: logistic regression; NB: naïve Bayes.The bolded values indicate the classifier models with the highest accuracy scores among the ones presented in each table.

## 4 Conclusion

In this paper, we have proposed an automated approach for the detection of ADHD using Gabor filter-based statistical features. Our methodology showed superior performance compared to the existing approaches to ADHD detection, signifying its potential as an efficient screening tool. However, this approach needs to be validated on a larger dataset before being used for any clinical purposes. As a part of our future study, we plan to explore deep-learning approaches for ADHD detection. Furthermore, we would like to explore Gabor filters for applications such as the classification of sleep stages and schizophrenia detection, which involves the classification of EEG signals.

## Data availability statement

The original contributions presented in the study are included in the article/supplementary material, further inquiries can be directed to the corresponding author.

## Author contributions

ES: Writing – original draft, Visualization, Validation, Software, Methodology, Investigation, Data curation, Conceptualization, Funding acquisition. TR: Writing – review & editing, Supervision, Funding acquisition, Formal analysis. TK: Writing – review & editing, Supervision, Investigation, Formal analysis.
